# Lineage‐specific epitope profiles for HPAI H5 pre‐pandemic vaccine selection and evaluation

**DOI:** 10.1111/irv.12466

**Published:** 2017-08-12

**Authors:** Xueting Qiu, Venkata R. Duvvuri, Jonathan B. Gubbay, Richard J. Webby, Ghazi Kayali, Justin Bahl

**Affiliations:** ^1^ Center for Infectious Diseases School of Public Health University of Texas Health Science Center Houston TX USA; ^2^ Public Health Ontario Toronto ON Canada; ^3^ University of Toronto Toronto ON Canada; ^4^ Mount Sinai Hospital Toronto ON Canada; ^5^ The Hospital for Sick Children Toronto ON Canada; ^6^ Department of Infectious Diseases St. Jude Children's Research Hospital Memphis TN USA; ^7^ Human Link Hazmieh Lebanon; ^8^ Program in Emerging Infectious Diseases Duke‐National University of Singapore Graduate Medical School Singapore Singapore

**Keywords:** HPAI H5, human CD8+ T‐cell epitope, vaccine selection

## Abstract

**Background:**

Multiple highly pathogenic avian influenza (HPAI) H5 viruses continue to co‐circulate. This has complicated pandemic preparedness and confounded effective vaccine candidate selection and evaluation.

**Objectives:**

In this study, we aimed to predict and map the diversity of CD8+ T‐cell epitopes among H5 hemagglutinin (HA) gene lineages to estimate CD8+ T‐cell immunity in humans induced by vaccine candidates.

**Methods:**

A dataset consisting of 1125 H5 HA sequences collected between 1996 and 2017 from avian and humans was assembled for phylogenetic and lineage‐specific epitope analyses. Conserved epitopes were predicted from WHO‐endorsed vaccine candidates and representative clade‐defining strains by pairwise comparison with Immune Epitope Database (IEDB). The distribution of predicted epitopes was mapped to each HPAI H5 lineage. We assume that high similarity and conservancy of predicted epitopes from vaccine candidates among all circulating HPAI H5 lineages is correlated with high immunity.

**Results:**

A total of 49 conserved CD8+ T‐cell epitopes were predicted at 28 different amino acid positions of the HA protein. Mapping these epitopes to the phylogenetic tree allowed us to develop epitope profiles, or “fingerprints,” for each HPAI H5 lineage. Vaccine epitope percentage analyses showed some epitope profiles were highly conserved for all H5 isolates and may be valuable for universal vaccine design. However, the positions with low coverage may explain why the vaccine candidates do not always function well.

**Conclusions:**

These findings demonstrate that our analytical approach to evaluate conserved CD8+ T‐cell epitope prediction in a phylogenetic framework may provide important insights for computational design of vaccine selection and future epitope‐based design.

## 1 INTRODUCTION

Highly pathogenic avian influenza (HPAI) H5 virus causes a highly infectious disease in birds and severe respiratory infections in humans.^1^ The HPAI H5N1 virus was first isolated from geese (prototype virus: A/goose/Guangdong/1/96) in China in 1996, and the first human infection occurred in Hong Kong in 1997.^2^ Since its emergence, HPAI H5N1 virus has rapidly evolved into at least ten co‐circulating lineages infecting many different host species.^3^

HPAI H5 is endemic in domestic poultry populations throughout China, South‐East Asia, and the Middle East with sporadic human outbreaks occurring in some countries.^4^ The virus has also re‐emerged numerous times, spilling over from wild birds to cause outbreaks in domestic populations in Europe, Africa, and North America.^4^, ^5^, ^6^, ^7^ Control of these outbreaks has resulted in hundreds of millions of domestic birds dying from illness or being culled.^8^, ^9^ Since 2003, the estimates of global loss from these outbreaks run into the billions of dollars.^9^, ^10^ In December 2014, a novel virus but carrying a different neuraminidase type (N2) containing the HA gene from HPAI H5N1 viruses was detected in North America leading to a widespread multistate outbreak among domestic poultry.^9^, ^11^ In addition to the risk of human infections and pandemic emergence, the loss of protein from decimated domestic flocks in H5 endemic regions is a severe threat to human health for resource‐challenged communities.^12^

A human‐adapted pandemic H5 virus may emerge, either through reassortment with circulating human‐adapted viruses or through evolutionary selection following repeated human infection. Even though human infection with HPAI H5 is relatively rare, when it does occur, the case fatality rate is high. From 2003 to 2014, 667 human cases have been identified, of which 58.9% were fatal.^13^ Although there is no evidence of sustained human‐to‐human transmission, the rapid emergence of novel strains of H5 viruses from animal reservoirs combined with the sporadic human infections indicates a high risk for pandemic HPAI H5 emergence. Two recent studies independently demonstrated that as few as three to five mutations in HPAI H5N1 viruses could allow for the effective aerosol transmission between ferrets.^14^, ^15^ Furthermore, about 50% of novel HPAI H5 strains generated from reassortment between contemporary avian H5N1 and human H3N2 showed a high degree of compatibility and potential virulence.^16^

Effective vaccines are important to mitigate the pandemic threat of HPAI H5 virus. For H5 virus, pre‐pandemic vaccine candidate strains are selected based on genetic surveillance data and comparative antigenic profiles.^17^ Viruses that stimulate broadly reactive antibodies against diverse strains should be ideal vaccine candidates.^18^ Highly effective vaccine candidates for HPAI H5 are difficult to predict due to co‐circulation of diverse lineages and genotypes.

One possible approach to develop highly cross‐reactive vaccine candidates for HPAI H5 is based on the cytotoxic T‐lymphocytes (CD8+ T cells), which tend to target more conserved influenza virus proteins (epitopes). This approach takes advantage of the memory CD8+ T cells that develop in response to vaccination or previous infection.^19^, ^20^ Evolutionarily conserved viral epitopes may be recognized by T cells or B cells/antibodies that have been previously primed by circulating influenza strains and can provide broad protection across different influenza types (also known as preexisting immunity).^21^ The H5 HA protein is a major surface protein that plays a pivotal role in viral infection and the primary immune target. Given the high diversity of HPAI H5 lineages, CD8+ T‐cell epitopes that are conserved across lineages may provide insights into whether or not a vaccine candidate may induce protection against other diverse or drifted HPAI viruses.

The CD8+ T‐cell responses are important for viral clearance, promoting disease recovery and reducing disease severity.^22^ Evidence from human^23^, ^24^ and animal^25^ studies shows that preexisting memory CD8+ T‐cell responses directed at conserved and/or cross‐reactive epitopes can prevent subjects from severe influenza infection and provide a measure of protection for the immune naive host.^24^ Although some epitope analyses, such as for H7N9,^26^ H3N2,^27^ and H1N1,^21^, ^26^, ^27^, ^28^ have been reported, no systematic studies of conserved HPAI H5 CD8+ T‐cell epitopes have been conducted.

In this study, we described the phylogenetic distribution of HPAI H5N1 epitopes binding to HLA‐A and recognized by human CD8+ T cells and developed lineage‐specific epitope profiles. Lineage‐specific epitope profiles based on similarity to vaccine candidate epitopes may be used to evaluate the vaccine candidates for their potential capability of inducing memory CD8+ T‐cell responses in humans. Furthermore, understanding the spatial and phylogenetic distribution of conserved and variable epitopes should be integrated into pre‐pandemic planning and vaccine selection.

## 2 METHODS

### 2.1 Dataset

HPAI H5N1 hemagglutinin (HA) gene nucleotide sequences of isolates with sampling dates from 1/1/1996 up to and including 3/31/2017 (Tables S1, S2) were downloaded from the *Global Initiative on Sharing All Influenza Data* (GISAID, www.gisaid.org) and *NCBI's GenBank* (http://www.ncbi.nlm.nih.gov/genbank/). The HA gene nucleotide sequences of HPAI H5 with other neuraminidase types, including H5N2, H5N6, and H5N8, were downloaded from 01/01/2014 to 3/31/2017, based on their emergence time and the appearance of multibasic cleavage sites. Criteria for selection of sequences included the following: (i) a known geographic location, host, and isolation year, (ii) sequences that were at least 1000 nucleotide bases in length. Exclusion criteria included the following: duplicate records or vaccine, derivative, and recombinant sequences. Nucleotide sequences were aligned using MUSCLE v3.8.^29^ After subsampling (criteria described in supplemental materials), a representative dataset was determined (n=1095; details in Table S3). In addition, we included 30 vaccine candidate strains selected by WHO for HPAI H5Nx^30^ in our final dataset (n=1125; full taxa in Fig. S2).

### 2.2 Conserved epitope prediction and Conservancy analysis

WHO vaccine candidates and clade‐defining reference strains of HPAI H5 viruses were used to predict all possible conserved virus epitopes recognized by human CD8+ T cells based on the availability of a database of previously identified epitope regions.^31^, ^32^ Beginning with the start codon (ATG), nucleotide sequences of these regions were translated into protein sequences in BioEdit v7.2.5.^33^ The protein sequences were uploaded into IEDB MHC‐I Binding Predictions (http://tools.immuneepitope.org/mhci/) to predict epitopes that bind to human class I peptide‐MHC supertypes. Conserved epitopes were predicted by searching the translated HA protein where each nine‐amino acid (aa) chain^34^ was compared to previously identified CD8+ T‐cell epitopes of influenza virus based on pairwise similarity.

Artificial neural network binding affinity (ANN IC_50_) is one of the indices to measure the computational affinity for HLA‐A of each epitope. Epitopes with IC_50_ less than 50 nmol/L were recognized as strong‐binding, while an IC_50_ range between 50 and 500 nmol/L was considered weak binding.^34^ For ease, epitopes with 9‐aa were named based on the position of the first amino acid (aspartic acid, translated from nucleotides GAT) in the HA protein chain after removing the signal peptide (ie, name corresponds to aa position 1‐552). The predicted epitopes were compared to the HA alignment of 1125 taxa dataset described above, in order to identify epitopes conserved among circulating H5 strains^35^ (http://tools.immuneepitope.org/tools/conservancy/iedb_input). From this analysis, conserved epitopes corresponding to the predicted 9‐aa epitope could be identified and mapped on to the estimated phylogeny.

### 2.3 Validation of predicted epitopes against experimentally defined epitope

The predicted CD8+ T‐cell epitopes of H5 HA protein in Table 2 column Epitope 1 (other mutants of epitopes in column Epitope 2‐5 were not included in this comparison analysis) were screened against the Immune Epitope Database (IEDB) (www.immuneepitope.org) repository through Influenza Research Database (https://www.fludb.org/), which contains the experimentally defined epitope information present in the published literature. Comparing CD8+ T‐cell epitopes against those experimentally confirmed epitopes would help in identifying those epitopes that are evolutionarily conserved. We assume that those epitopes that are conserved across lineages will induce some degree of immune protection against various lineages.

### 2.4 Phylogenetic and coalescent analysis

The final dataset of 1125 nucleotide sequences of the HA gene were used to reconstruct the phylogeny of HPAI H5 with both maximum‐likelihood (ML) and Bayesian phylogenetic methods. The ML phylogenetic tree was performed with RAxML using the GTR‐GAMMA model.^36^ No bootstrap analysis was conducted. The best‐scoring ML tree was automatically generated from three runs by RAxML, and visualized in FigTree v1.4.2 (http://tree.bio.ed.ac.uk/software/figtree/) and rooted to A/goose/Guangdong/1/96 (AF148678).

The Bayesian phylogenetic analysis was performed using a lognormal relaxed molecular clock in a Bayesian statistical framework implemented in BEAST v.1.8.4.^37^ Molecular clock rates were uncorrelated with an initial mean of 0.0033 with a uniform prior ranging from 0.0 to 1.0. An HKY85^38^ nucleotide substitution model and a constant coalescent prior^39^ were used. Three independent Markov chain Monte Carlo (MCMC) simulations of 100 million generations were analyzed. The runs were combined after removing an appropriate burn‐in; that is, samples at the beginning of the MCMC run prior to its convergence to the stationary distribution were discarded. TreeAnnotator version 1.8.4 (http://beast.bio.ed.ac.uk/) was used to generate the maximum clade credibility (MCC) phylogenetic tree, which was visualized in FigTree v1.4.2. The time of most recent common ancestor (TMRCA) and 95% BCI (Bayesian credible interval) was determined for important nodes of different clades. To clearly show the currently circulating strains on the BEAST phylogenetic tree, timescale was set with label spacing as 5 years and tick spacing as 0.5 years.

### 2.5 Conserved epitope mapping

The distribution of conserved CD8+ T‐cell epitopes across all HPAI H5 isolates was mapped onto the ML tree by new grouped clades (S‐part II). The percentages of conserved epitopes presented in each clade were calculated and displayed as a pie graph for each epitope position. For diverse epitopes in one position, the percentage of each epitope was calculated, and the pie graph contained different colors to represent different epitopes in Table 2.

We defined the isolates from 01/01/2012 to 03/31/2017 as currently circulating strains. To understand the distribution of conserved CD8+ T‐cell epitopes in these circulating strains at different geographic locations, a similar strategy of epitope mapping as above was used. Only the percentages of conserved epitopes were calculated for each geographic location, not for each clade.

### 2.6 Clade‐specific epitope profile

Clade‐specific epitope profiles were calculated to examine how effectively the vaccine candidates can simulate conserved CD8+ T‐cell epitopes across the diverse HPAI H5 viruses. For this analysis, we assume that high similarity and conservancy of predicted epitopes from vaccine candidates among all HPAI H5 lineages is correlated with high immunity. Thirty vaccine candidates covered clades 1, 2.1, 2.2, 2.3, 4, and 7; therefore, epitope profiles were created for these groups. Conserved epitopes in one specific vaccine strain were examined to determine the degree of similarity of all HPAI H5 isolates in the vaccine‐corresponding clade. Clade‐specific epitope profile results were reported as the proportion of H5 strains in each clade that were resembled by each epitope in this vaccine candidate. For ease, individual proportion for each epitope in the vaccine candidates was shown as a heat map. Mean epitope coverage, as the measure for overall capability of one vaccine candidate to induce CD8+ T‐cell immunity in humans, was calculated by averaging the proportions across all epitope positions.

Further, the epitope profile was created for currently circulating H5 strains. The same strategy as above was employed, but the denominator for the proportion is the number of currently circulating strains in each clade. The vaccine candidates selected for clades no longer circulating were not included in the epitope profile analysis. The heat map of epitope profile for currently circulating strains is displayed in Fig. S1.

## 3 RESULTS

### 3.1 Phylogenetic structure and evolutionary history of HPAI H5

**Figure 1:**
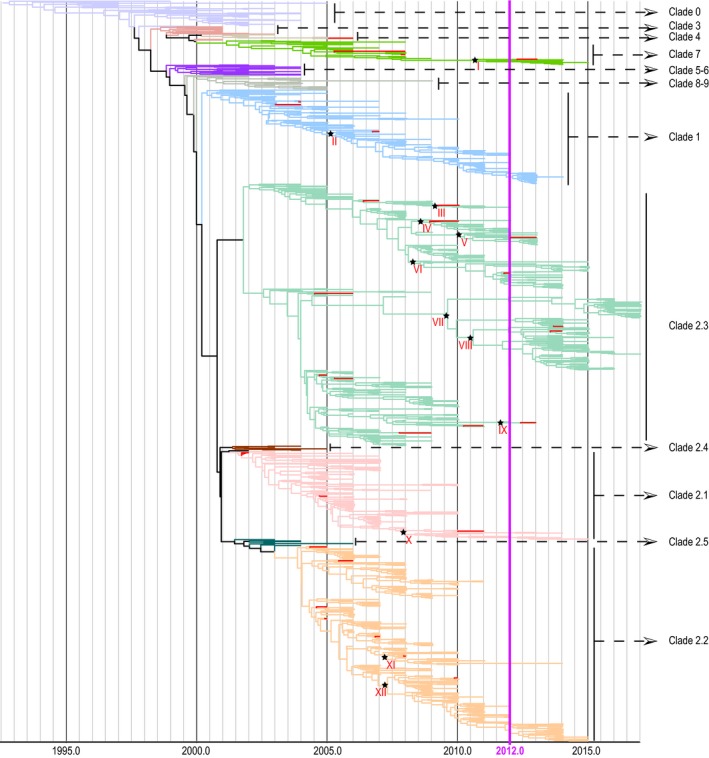
Bayesian relaxed clock phylogenetic tree highlighting HPAI H5 strains isolated since 2012. Red branches represent vaccine candidates. Strains isolated since 2012 are defined as currently circulating strains in this study. Tree nodes annotated with stars are the most common ancestor between currently circulating strains and their closest vaccine candidates. TMRCAs were shown in Table 3

Bayesian simulation of phylogenetic history using 1125 full‐length H5 HA nucleotide sequences (Figure 1) showed lineage diversification consistent with the WHO/OIE/FAO H5N1 classification system.^40^ The major clades, such as clade 1, 2.1, 2.2, 2.3, and 7, persisted through to the most recent sampling date included. A number of smaller clades did not persist and became extinct before 2010. The time of most recent common ancestors (TMRCA) for circulating HPAI H5 clades is presented in Table 1.

**Table 1:** Estimated TMRCAs for major HPAI H5 clades

Clades	Mean TMRCA^a^	95% BCI^a^
Clade 0 (Overall H5 ancestor)	1992.82	1989.77‐1994.91
Clade 1	2000.20	1999.73‐2000.66
Clade 2	2000.78	2000.37‐2001.18
Clade 2.1	2001.57	2001.23‐2001.85
Clade 2.2	2003.87	2003.50‐2004.24
Clade 2.3	2001.79	2001.24‐2002.28
Clade 2.4	2001.38	2000.98‐2001.76
Clade 2.5	2001.81	2001.35‐2002.21
Clade 3	1998.62	1998.00‐1999.16
Clade 4	2001.45	2000.39‐2001.57
Clade 5‐6	1998.83	1998.23‐1999.36
Clade 7	2001.60	2000.28‐2001.90
Clade 8‐9	1999.89	1999.39‐2000.38

a The dates are presented as decimal years.

TMRCA, the time of most recent common ancestor; 95% BCI, 95% Bayesian credible interval.

Contemporary strains, defined as those isolated between 01/01/2012 and 3/31/2017, comprised 253 isolates. They were found in six different regions, including East Asia (China, Japan, and South Korea), Europe (Austria, Netherlands, Romania, Sweden, UK, Bulgaria, Germany, Croatia, Italy, Poland, Republic of Czech, and Russia), Middle East (Egypt and United Arab Emirates), North America (the USA and Canada), South Asia (Nepal and Bangladesh), and South‐East Asia (Vietnam, Cambodia, and Indonesia). The majority (68.37%) of strains isolated from the main HPAI H5 epidemic centers in Asia, which included East Asia (36.36%), South‐East Asia (25.69%), and South Asia (6.32%). The majority (69.96%) of these currently circulating isolates belonged to clade 2.3, which showed a “bushy” tree pattern, suggesting multiple co‐circulating lineages and persistent viral diversity (Figure 1). Fewer (14.62%) belonged to clade 2.2, which displayed a ladderlike pattern, suggesting that most variants did not persist in the populations or regions surveyed. While 9.49% of these isolates belonged to clade 1, the remainder belonged to clades 7 (3.56%) and 2.1 (2.37%).

### 3.2 Prediction of conserved CD8+ T‐cell epitopes

Based on the 30 vaccine candidates selected by the WHO (Table S4) and 28 clade‐defining strains (Table S5) of HPAI H5, 49 CD8+ T‐cell epitopes (Table 2) binding to 28 different regions on the HA protein were predicted. Among these 28 positions, 18 had only one epitope identified, while the other 10 positions presented diverse epitopes. Positions 43, 103, 375, and 537 contained two distinct epitopes; positions 197, 389, 531 consisted of three epitopes; position 527 had four epitopes; and positions 78 and 529 each contained five epitopes.

**Table 2:** Strong‐ and weak‐binding HPAI H5 epitopes included in this study

Position of starting aa^a^	Epitope 1	Epitope 2	Epitope 3	Epitope 4	Epitope 5
>Position18	**TIMEKNVTV**				
>Position72	NVPEWSYIV				
>Position139	GMPSFFRNV				
>Position224	GRMEFFWTI				
>Position225	RMDFFWTIL				
>Position312	VLATGLRNA				
>Position332	GLFGAIAGF				
>Position345	GMVDGWYGF				
>Position414	KMEDGFLDV				
>Position419	**FLDVWTYNA**				
>Position431	VLMENERTL				
>Position438	TLDFHDSNV				
>Position515	YQILSIYST				
>Position516	QILSIYSTV				
>Position517	ILSIYSTVA				
>Position523	TVASSLALA				
>Position532	IMMAGLSLW				
>Position537	FLWMCSNGS				
>Position43	NLDGVKPLI	SLDGVKPLI			
>Position103	HLLSRINHL	HLLNRITHL			
>Position375	AIDGVTNKV	AIDGITNKV			
>Position533	**MMAGLSLWM**	MIAGLSLWM			
>Position197	YISVGTSTL	YISIGTSTL	YVSIGTSTL		
>Position389	**KMNTQFEAV**	KMNTQFEIV	KMNTQFEAI		
>Position531	AIMMAGLSL	AIMVAGLFL	AIMVAGLSL		
>Position527	SLALAIMVA	SLALAIMMA	SLALAIMIA	SLVLAIMVA	
>Position78	YIVEKASPA	**YIVEKANPV**	YIVEKANPA	**YIVEKINPA**	**YIVEKTNPA**
>Position529	ALAIMVAGL	ALAIMMAGL	**ALAIMIAGL**	VLAIMVAGL	VLAIMMAGL

a Conventionally, the position of starting amino acid along HA protein sequence after removing the signal peptide was used to identify the epitope.

Bold and underlined format represents strong‐binding epitope. The epitope in each column was coded as “1,” “2,” “3,” “4,” “5,” accordingly. For example, in the column “EPITOPE 2,” if epitope *SLDGVKPI* was present in position 43 in a certain H5N1 strain, then it was coded as “2” for position 43.

The study aimed to provide information for vaccine development, and the number of conserved viral epitopes predicted to react with CD8+ T cells was specifically displayed for each vaccine candidate and its clade category (Table S4). The 30 vaccine candidate strains selected by WHO covered clades 1, 2.1, 2.2, 2.3, 4, and 7 (Table S4, Figure 1) with most of the strains belonging to clade 2.2 and clade 2.3. The number of conserved epitopes in each vaccine strain was similar across different clades; that is, each vaccine candidate contained 13‐21 conserved viral epitopes recognized by CD8+ T cells with two to four strong‐binding epitopes. In contrast, the number of weak‐binding epitopes was approximately three to five times that of the strong‐binding epitopes for each vaccine. Importantly, the epitopes that we identified could be validated through comparison with experimentally identified CD4+ T‐cell epitopes, revealing that 43% (12/28) of our predicted CD8+ T‐cell epitopes in Table 2 column Epitope 1 were conserved with previously identified CD4+ T‐cell epitopes (Table S6).

### 3.3 Epitope distribution across all H5 clades on phylogenetic tree

**Figure 2:**
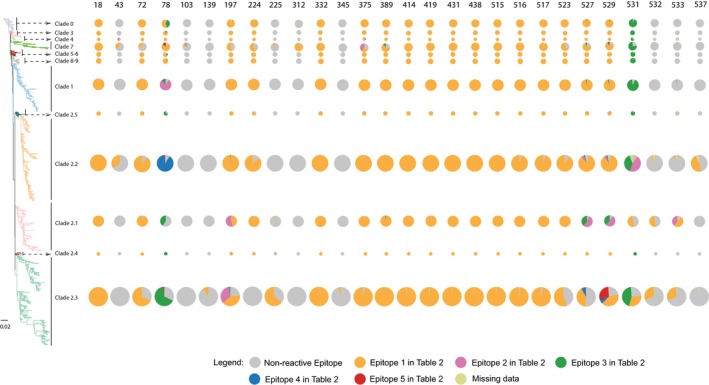
Maximum‐likelihood phylogenetic tree and the distribution of CD8+ T‐cell epitopes across lineages. The tree is rooted with the earliest isolate, goose/Gd/1/96. Epitopes corresponding to those identified in Table 2 are mapped to the leaves of each lineage. Rows show the variability of CD8+ T‐cell epitopes in each lineage, and columns correspond to position along HA protein sequence. The size of pie for each clade is proportional to the number of isolates in each clade. Color legends for the pies are displayed in the figure. To help read the figure, for example, orange color represents Epitope 1 in Table 2 for each position

The distribution of CD8+ T‐cell epitopes across all HPAI H5 clades was mapped to the tips of the ML phylogenetic tree (Figure 2). The distribution of conserved epitopes significantly varied among HPAI H5 clades. Three general phenomena were observed. First, positions 18, 332, and 414‐522 were conserved across all lineages with almost 100% of sampled isolates possessing the same epitopes as the vaccine strain. In contrast, positions 43, 103, 139, 312, 345, 532, and 537 had very low percentages of sampled isolates covered by the predicted epitopes, and those epitopes only occurred in certain clades across the phylogeny, which may be interpreted as clade‐specific “fingerprints.” Finally, positions 78, 197, 527, 529, 531, and 533 were highly diverse across the phylogeny with multiple epitopes present. The diverse epitopes in one position may only present in one or two specific clades, not across all clades. They form specific epitope profile for each clade. For example, position 531 Epitope 3 *AIMVAGLSL* (Figure 2; shown in green) was present in clades 0‐9, clade 2.4, and the majority of clade 2.3, while Epitope 2 *AIMVAGLFL* (Figure 2; shown in purple) was seen only in clade 2.2, and Epitope 1 *AIMMAGLSL* (shown in orange) was mainly in clade 2.1 and 2.3.

### 3.4 Epitope diversity and distribution among currently circulating H5 strains

**Figure 3:**
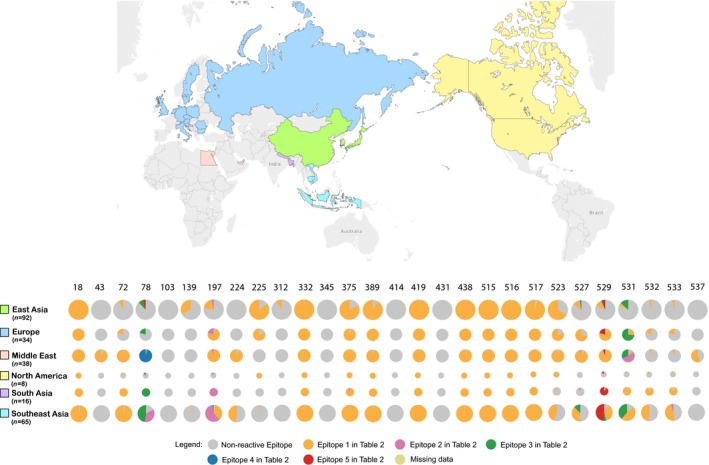
Geographic distribution of recent HPAI H5 strains and conserved CD8+ T‐cell epitopes. The rows show the distribution of CD8+ T‐cell epitopes by locations, and columns show variability in epitope positions along HA protein sequence. The size of pie for each geographic location is proportional to the number of isolates in each region. Color legends for the pies are displayed in the figure. To help read the figure, for example, orange color represents Epitope 1 in Table 2 for each position

Multiple co‐circulating HPAI H5 virus strains confound disease control and pandemic preparedness efforts. We examined the spatial distribution of CD8+ T‐cell epitopes among currently circulating strains as a proxy measurement of within‐region viral diversity and to identify populations where the selected vaccine candidates may be less effective (Figure 3). Epitope profiles conserved across all locations and location‐specific profiles were both observed. In all geographic locations, epitopes at positions 103, 139, 312, 345, 414, and 431 were judged non‐reactive, whereas positions 18, 72, 332, 389, 414, 419, 431, 438, 515, 516, and 517 had single reactive epitope. Geographic locations differed for some positions which contained a single epitope unique to that location: positions 43 and 537 were only found in Middle East; position 224 was found in Middle East and South‐East Asia; position 312 was only found in East Asia; position 523 and 532 had single epitope presenting in all regions but not all isolates contained the epitope. The distribution of multiple epitopes in positions 78, 197, 375, 527, 529, 531, and 533 revealed both epitope‐specific and location‐specific features.

### 3.5 Epitope profile of clade‐specific vaccine candidates

**Figure 4:**
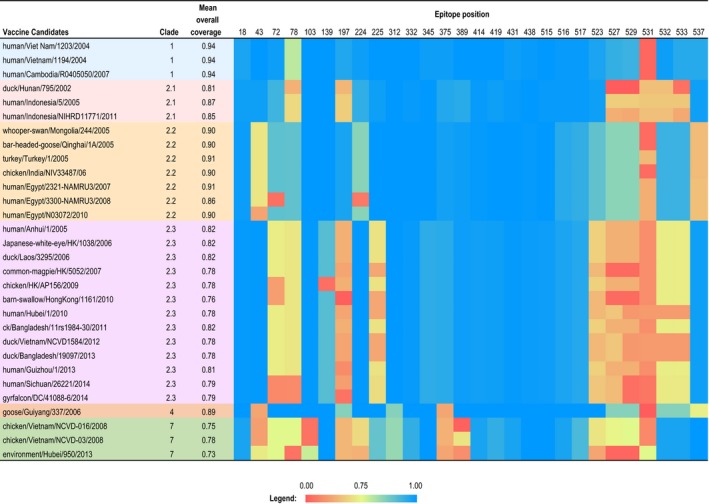
Heat map showing homology to epitope profiles predicted from clade‐specific vaccine candidates among contemporary HPAI H5 circulating viruses. Clade‐specific epitope profile reported as the proportion of H5 strains in each clade with high similarity to epitope profiles of the vaccine candidate. The color range represents the proportion from 0.00 to 1.00 in the heat map. The mean epitope coverage was calculated from averaging the proportions in different epitope positions

Epitope profiles were examined for HPAI H5 isolates to estimate the capability of each vaccine candidate to induce immunity. All 30 vaccine candidates were compared to all isolates to identify homologous epitope profiles in each corresponding clade (Figure 4). For 29 vaccines candidates of clades 1, 2.1‐2.3, and 7, the profile was calculated only for currently circulating strains (Fig. S1).

The majority of epitope positions in each vaccine candidate had very high proportions (blue, Figure 4) that simulate all clade‐specific H5N1 isolates, whereas each vaccine candidate contained positions with very low proportion (red and orange, Figure 4). The mean epitope coverage of three vaccine candidates from clade 1 was 0.94, with very low proportion in position 531, where the vaccine candidates simulate only 1% of H5 clade 1 isolates. For three strains of vaccine candidates from clade 2.1, the mean coverages were 0.81, 0.87 and 0.85, with positions 78, 197, 527‐533 as low proportion. The mean coverage of seven vaccine candidates from clade 2.2 was about 0.90, and positions 43, 72, 224, 531, and 537 had low proportion. Vaccine candidates in clade 4 had similar profile with these in clade 2.2, but positions 43, 375, 531, and 537 had low proportion. Vaccine candidates from clades 2.3 and 7 had similar mean coverages, which was about 0.80 and obviously lower than the vaccine candidate mean coverage in other clades. Each vaccine candidate in these two clades generally had more positions covering only few of the isolates in their clades.

Further epitope profile analysis for currently circulating H5 strains showed similar results (Fig. S1). Specifically, there were more extreme low values (0 or 0.01), indicating the vaccine candidates may be less effective for currently circulating H5 isolates. Three clade 1 vaccine candidates with mean coverage of 0.95 had generally high proportions across 27 viral epitope positions (except for position 531), suggesting these vaccine strains may be good candidates to provide broad cross‐reactive protection. Vaccines in clade 2.3 and 7 had lower mean coverage (0.73‐0.84), indicating the virus isolates were more diverse where some of these candidates may not be representative of the epitope profile for other circulating variants.

To connect epitope profile with phylogenetic information, the TMRCAs between the currently circulating strains and their closest vaccine candidate (Table 3) were reported for each clade based on the Bayesian phylogenetic tree (black stars, Figure 1). Generally, the majority of TMRCAs were close to recent years ranging from January 2008 to October 2011, except for clade 1 with the TMRCA as March 2005. Even though the vaccine candidates in clades 1, 2.1, and 2.2 were selected from early year isolates, they had a high mean coverage around 0.90 (the highest as 0.95), which indicated that these clades remained highly conserved throughout their evolutionary history. For clade 7, the vaccine candidate has TMRCA from 2011 and the mean coverage is 0.88. For clade 2.3, the mean coverages of the vaccine candidates were generally low, no matter when the vaccine candidates were selected. The two 2014 vaccine candidates for clade 2.3 on nodes VII and VIII had 84% coverage.

**Table 3:** The divergence times estimated between currently circulating HPAI H5 strains and their closest vaccine candidate

Nodes	Vaccine candidate	MC	Clade	Mean TMRCA^a^	95% BCI^a^
I	Environment/Hubei/950/2013	0.88	7	2011.18	2009.66‐2011.65
II	Human/Cambodia/R0405050/2007	0.95	1	2005.21	2004.85‐2005.36
III	Barn‐swallow/HK/1161/2010	0.76	2.3	2009.26	2008.80‐2009.70
IV	Human/Hubei/1/2010	0.77	2.3	2008.65	2008.17‐2009.10
V	Duck/Bangladesh/19097/2013	0.77	2.3	2010.10	2009.60‐2010.54
VI	Duck/Vietnam/NCVD1584/2012	0.77	2.3	2008.58	2008.26‐2008.85
VII	Human/Sichuan/26221/2014	0.84	2.3	2009.62	2008.67‐2010.57
VIII	Gyrfalcon/DC/41088‐6/2014	0.84	2.3	2010.60	2009.77‐2011.36
IX	Human/Guizhou/1/2013	0.79	2.3	2011.77	2011.28‐2012.04
X	Human/Indonesia/NIHRD11771/11	0.95	2.1	2008.01	2007.70‐2008.30
XI	Human/Egypt/3300‐NAMRU3/2008	0.85	2.2	2007.31	2007.07‐2007.53
XII	Human/Egypt/N03072/2010	0.95	2.2	2007.32	2006.96‐2007.72

Nodes: Ancestral nodes between currently circulating strains and their closest vaccine candidate, marked as black stars in Figure 1.

MC, Mean overall coverage for each vaccine candidate from Fig. S1.

a The dates are presented as decimal years.

TMRCA, the time of most recent common ancestor; 95% BCI, 95% Bayesian credible interval.

## 4 DISCUSSION

In this study, we estimated the diversity and spatial distribution of HPAI H5 epitopes binding to HLA‐A and recognized by human CD8+ T cells. Phylogenetic analysis of HPAI H5 showed multiple lineages are co‐circulating in numerous countries. The distribution of HPAI H5 conserved epitopes mapped onto a phylogenetic tree allowed us to generate lineage‐specific and location‐specific epitope profiles for currently circulating strains. We used these profiles to estimate the capability of vaccine candidate viruses to induce CD8+ T‐cell response in humans, suggesting that highly conserved epitopes may be valuable for universal vaccine design and some vaccine candidates might perform poorly to protect against the high diversity of contemporary viruses circulating.

HPAI H5 viruses have circulated continuously for two decades, diversifying to several persisting clades (1, 2.1, 2.2, 2.3, and 7), primarily isolated from Asian countries. The high density of poultry and humans, the asymptomatic carriage among migratory wild birds, and shared habitats/housing of poultry and waterfowl combined with poor agricultural vaccines have contributed to the diversification and persistence of HPAI H5.^12^, ^41^ The potential severity of an H5 pandemic remains a major concern.^42^ One strategy to mitigate large‐scale loss of life is to stockpile effective vaccine candidates that can be deployed in the event of an H5 pandemic occurrence. Combining our phylogenetic analysis with lineage‐specific mapping of CD8+ T‐cell epitopes across all HPAI H5 clades provides a unique CD8+ T‐cell epitope fingerprint that allows for important conserved characteristics to be easily identified. In addition, data generated through genetic surveillance may be utilized for crude, but rapid predictions of effective vaccine candidates against emerging lineages and pandemic risk assessment.

An ideal pre‐pandemic vaccine candidate strain would contain all important characteristics of specific strains to be effective, yet still share enough characteristics with other circulating viruses to induce broad protection against as many potentially pandemic viruses as possible.^43^ CD4+/CD8+ T‐cell epitopes represent heritable phenotypic characteristics that are recognized by the host immune system. CD8+ T‐cell epitopes for internal genes and CD4+ T‐cell epitopes for HA were mostly studied for epitope‐based vaccine design.^19^, ^22^, ^44^, ^45^ Studies showed that influenza HA does have conserved CD8+ T‐cell epitopes.^21^, ^46^ While our results have focused on the predicted CD8+ T‐cell epitopes, a high proportion of the predicted epitopes overlapped with experimentally confirmed CD4+ T‐cell epitopes. Overlapping CD8+ and CD4+ T‐cell responses to conserved epitope may significantly influence the course of influenza infection, as the presence of CD4+ T‐cell responses can greatly enhance and amplify the immune response of memory CD8+ T cells, and regulate the production of neutralizing antibodies from B‐lymphocytes.^47^

These types of conserved and epitope‐specific cross‐immune response become crucial, when a novel strain appears in the absence of preexisting antibodies. We found that one predicted CD8+ T‐cell epitope (GLFGAIAGF) is commonly conserved across the subtypes of H1N1 (seasonal 1977‐2009 and 2009 pandemic), H2N2, H5N1, H7N7, and H7N9; and the previously published experimental studies reported that GLFGAIAGF has potential to induce B‐ and T (CD8+ and CD4+) immune responses. This identified epitope could be useful in designing treatments that offer heterosubtypic protection. Evolutionarily conserved and cross‐reactive T‐cell epitopes in the HA protein further underpin the importance of systematic epitope investigations in the development of epitope‐based vaccine design.

For the evaluation of immunity induction of vaccine candidates selected by the WHO, our results of highly conserved epitopes indicate potential cross‐reactivity of vaccine candidates to circulating viruses in other clades. In our analysis, assuming that high similarity and conservancy of predicted epitopes from vaccine candidates among all HPAI H5 lineages is correlated with high immunity, we can predict that vaccine candidates in clades 2.3 and 7 might not stimulate broadly reactive immune protection. Vaccine candidates are often selected based on antigenic divergence from the population mean.^48^ However, the rate of antigenic divergence is a function of epidemic size, duration of outbreaks, and geographic structuring of lineages.^48^, ^49^ In addition, the long‐term circulation of some lineages may result in older vaccine candidates stimulating less specific epitope coverage for currently circulating strains due to genetic drift.^50^ As a result, it is not surprising that lineage or geographic specific vaccine candidates may be integrated in to the pre‐pandemic candidate virus stockpile. This may explain why qualitatively the vaccine candidates for clades 2.3 and 7 generally presented lower epitope mean coverage than other candidates. While epitopes highly conserved between lineages may indicate higher vaccine effectiveness based on our assumption, the lack of experimental data limits our ability to make confident predictions. Integrating epitope profiles with experimentally confirmed epitopes, antigenic characterizations, and comparative genetic analysis may improve such predictions.

This study is the first comprehensive prediction of conserved CD8+ T‐cell epitopes mapped on phylogeny and the evaluation of possible immunity induction of vaccine candidates conducted for HPAI H5 viruses. However, HPAI H5 epitope predictions based on publicly available genetic data may have sampling biases.^51^ In this study, sampling biases were managed by randomly subsampling available data to balance the contribution of outbreaks from different locations and years. In addition, the prediction of human epitopes is based on vaccine candidates that have primarily been collected from avian hosts. Host adaptation mutations, including silent mutations associated with changes in codon usage bias, have not been considered in our epitope prediction. Moreover, an analysis focused only on HA protein has limitations.^52^ Many studies^19^, ^52^ reported that the immunodominance of T cellular responses from the T‐cell epitopes derived from internal proteins, as they are highly conserved. Hence, to identify all potential vaccine candidate epitopes (from all types of proteins) for HPAI H5, it would be worth conducting further studies that consider the inductive potential of other viral proteins. Even though conserved CD8+ T‐cell epitopes were predicted from genetic motifs, which may provide insight into developing a universal vaccine, inaccuracies from epitope prediction programs may generate misleading results that need to be confirmed experimentally.^43^, ^53^ A clear limitation of sequence‐based inference is that the utility of these highly conserved epitopes to induce broadly protective immunity needs to be experimentally tested. Studies in animal models or in vitro human CD8+ T cells are necessary and will help confirm the true immunogenic potential of HA epitopes.

Currently, commercial inactivated whole‐virus vaccines are produced by exposing the virus of interest to a cross‐linking agent such as formaldehyde or an alkylating agent such as beta‐propiolactone. Incorporating the diversity of predicted epitopes may be useful for computationally optimized design of broadly cross‐reactive vaccine candidates including the development of T‐cell epitope‐based vaccines.^54^ With experimental confirmation of true immunogenic CD8+ T‐cell epitopes, the spatial and phylogenetic distribution of conserved and variable epitopes should be integrated into pre‐pandemic planning and vaccine selection to ensure broad reactivity and potential effectiveness. The frequency of expression of different HLA alleles varies across different ethnicities.^55^ Future studies integrating HLA allele frequency, host demographics, epitope predictions, whole viral proteome, and viral genomic diversity into computational models may provide novel insights into vaccine effectiveness and risk assessment of potential pandemic.

## CONFLICT OF INTEREST

Jonathan Gubbay (co‐author) has received research grants from GlaxoSmithKline Inc. and Hoffmann‐La Roche Ltd. to study antiviral resistance in influenza, and from Pfizer Inc. to conduct microbiological surveillance of Streptococcus pneumoniae. These activities are not relevant to this study.
