# Supplementary material for: Lineage‐specific epitope profiles for HPAI H5 pre‐pandemic vaccine selection and evaluation
Source: Influenza Other Respir Viruses. 2017 Aug 12;11(5):445–56. doi: 10.1111/irv.12466 (PMC5963872; doi:10.1111/irv.12466)
Supplement: Supplementary file 1 [file IRV-11-445-s001.pdf]

Figure S1. Heat map predicting the capability of vaccine candidates to induce preexisting immunity in humans for currently circulating HPAI H5 strains

Vaccine Candidates	Clade	Mean overall coverage
human/Viet Nam/1203/2004	1	0.95
human/Vietnam/1194/2004	1	0.95
human/Cambodia/R0405050/2007	1	0.95
duck/Hunan/795/2002	2.1	0.82
human/Indonesia/5/2005	2.1	0.80
human/Indonesia/NIHRD11771/2011	2.1	0.95
whooper-swan/Mongolia/244/2005	2.2	0.89
bar-headed-goose/Qinghai/1A/2005	2.2	0.89
turkey/Turkey/1/2005	2.2	0.90
chicken/India/NIV33487/06	2.2	0.89
human/Egypt/2321-NAMRU3/2007	2.2	0.92
human/Egypt/3300-NAMRU3/2008	2.2	0.85
human/Egypt/N03072/2010	2.2	0.95
human/Anhui/1/2005	2.3	0.79
Japanese-white-eye/HK/1038/2006	2.3	0.79
duck/Laos/3295/2006	2.3	0.79
common-magpie/HK/5052/2007	2.3	0.76
chicken/HK/AP156/2009	2.3	0.77
barn-swallow/HongKong/1161/2010	2.3	0.76
human/Hubei/1/2010	2.3	0.77
ck/Bangladesh/11rs1984-30/2011	2.3	0.79
duck/Vietnam/NCVD1584/2012	2.3	0.77
duck/Bangladesh/19097/2013	2.3	0.77
human/Guizhou/1/2013	2.3	0.79
human/Sichuan/26221/2014	2.3	0.84
gyrfalcon/DC/41088-6/2014	2.3	0.84
chicken/Vietnam/NCVD-016/2008	7	0.66
chicken/Vietnam/NCVD-03/2008	7	0.66
environment/Hubei/950/2013	7	0.88

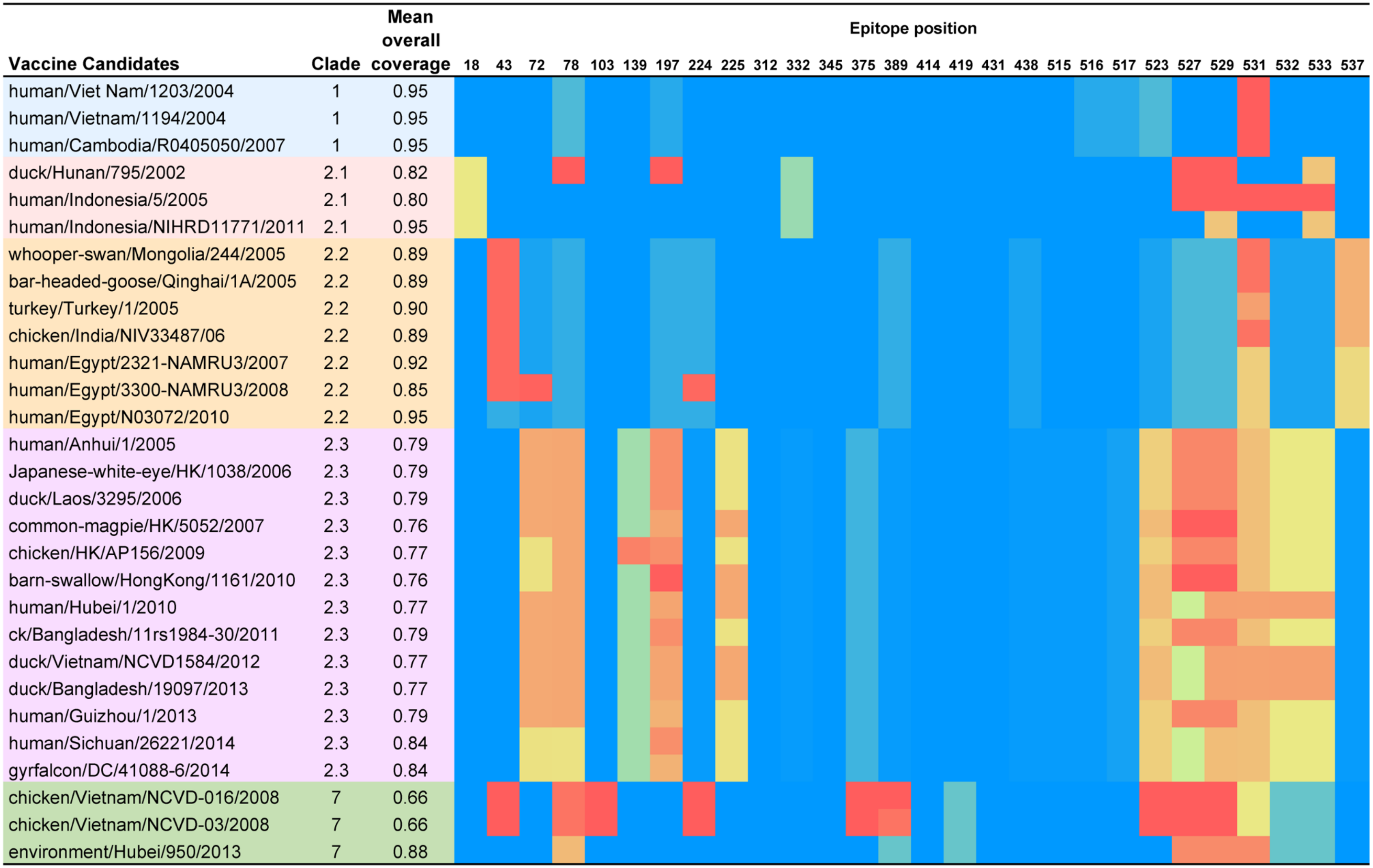

Legend:
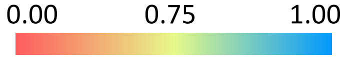

Clade-specific epitope profile was reported as the proportion of H5 strains in each clade that were resembled by each epitope in this vaccine candidate. Mean epitope coverage, as the measure for overall potential of one vaccine candidate to induce preexisting immunity in humans, was calculated from averaging the proportions in different epitope positions.
