# Supplementary material for: Lineage‐specific epitope profiles for HPAI H5 pre‐pandemic vaccine selection and evaluation
Source: Influenza Other Respir Viruses. 2017 Aug 12;11(5):445–56. doi: 10.1111/irv.12466 (PMC5963872; doi:10.1111/irv.12466)
Supplement: Supplementary file 3 [file IRV-11-445-s003.docx]

**Supplemental materials**

**S-Part I.**

**Data subsampling:**

To describe the dataset and estimate viral histories, taxa were coded by clades, isolation year and host species (i.e. avian or human). The clades are classified by the nomenclature system proposed by the WHO/OIE/FAO H5N1 Evolution Working Group. Isolates (n=4,147) were stratified by year of collection, source and clade to characterize the final dataset and identify potential sources of sampling biases (Table S1, S2). Since the sampled isolates are highly related to the scale of each single outbreak, regardless of the disease prevalence in the community, sampling biases exist. Therefore, the sample size of each category by clades and years that had twenty or more isolates (highlighted as red in Table S2) was randomly reduced to twenty to balance the contribution of each category to the whole date set. The whole dataset was then randomly reduced by removing the strains with the same location and date, only one from which was kept to ensure that different surveillance resources and efforts at different regions did not contribute to sampling bias in the study. The distribution of the final dataset is shown in Table S3.

**S-Part II.**

**New grouped HPAI H5N1 clades:**

The NJ tree and clade classification from WHO/OIE/FAO H5N1 Evolution Working Group were compared to the ML Phylogenetic tree. New groups of H5 clades were created based on the ML phylogenetic tree to combine genetic subclades into one group, since epitopes are usually very conserved within clades and the data for epitope mapping is not informative at subclade level. The originally classified 2^nd^, 3^rd^, and 4^th^ order of clades were grouped based on their first order classification (clade 0, 1, 3-9), with the exception of clade 2 where the 2^nd^ order groupings (clades 2.1 -2.5) were retained. These groups were highly divergent and well sampled to justify this treatment of clade 2. Clades with a small number of H5 isolates and without clade-specific vaccine strains were grouped as follows: the original clade 5, clade 5-6-like, and clade 6 were renamed as clade 5-6; clade 8, clade 8-9-like, and clade 9 isolates were grouped as clade 8-9; clade 1-8-9-like was classified as clade 1 since it was located closer to clade 1 than to clade 8-9; and 3 strains of clade 2-like were incorporated into clade 2.5. To this end, twelve (12) new phylogenetic groups for this study were formed, which were displayed by names at the right of the ML and BEAST tree (Figures 1, 2).

Table S1. The number of publicly available HPAI H5 sequences from GenBank and GISAID

| Year |  | Avian | Humans | Environment | TOTAL |
| --- | --- | --- | --- | --- | --- |
| 1996 |  | 1 | 0 | 0 | **1** |
| 1997 |  | 15 | 14 | 2 | **31** |
| 1998 |  | 0 | 1 | 0 | **1** |
| 1999 |  | 3 | 0 | 4 | **7** |
| 2000 |  | 21 | 0 | 0 | **21** |
| 2001 |  | 45 | 1 | 0 | **46** |
| 2002 |  | 56 | 0 | 1 | **57** |
| 2003 |  | 86 | 8 | 0 | **94** |
| 2004 |  | 202 | 25 | 0 | **227** |
| 2005 |  | 342 | 31 | 1 | **374** |
| 2006 |  | 503 | 87 | 2 | **592** |
| 2007 |  | 486 | 40 | 6 | **532** |
| 2008 |  | 274 | 20 | 3 | **297** |
| 2009 |  | 202 | 36 | 13 | **251** |
| 2010 |  | 244 | 17 | 4 | **265** |
| 2011 |  | 283 | 32 | 4 | **319** |
| 2012 |  | 75 | 5 | 0 | **80** |
| 2013 |  | 48 | 15 | 9 | **72** |
| 2014 |  | 321 | 13 | 40 | **374** |
| 2015 |  | 288 | 9 | 82 | **379** |
| 2016 |  | 70 | 2 | 3 | **75** |
| 2017 |  | 52 | 0 | 0 | **52** |
| TOTAL |  | **3617** | **356** | **174** | **4147** |

Table S2. Frequencies of HPAI H5 HA gene sequences by hosts, years and clades highlighting cells that contain ≥ 20 strains

Notes: highlighted cells were subsampled.

Table S3. Frequencies of HPAI H5 HA gene sequences by hosts, years and clades after subsampling

Note: n=1095 sequences were retained after subsampling. An additional 30 vaccine candidates were included in the final dataset (n=1125).

Table S4. Epitope conservancy analysis of 30 HPAI H5 vaccine candidates

| Vaccine Candidates | Clade | No. of CD8+ T-cell epitopes | | |
| --- | --- | --- | --- | --- |
|  |  | **Total** | **Strong-Binding** | **Weak-Binding** |
| human/Viet Nam/1203/2004 | 1 | 18 | 4 | 14 |
| human/Vietnam/1194/2004 | 1 | 18 | 4 | 14 |
| human/Cambodia/R0405050/2007 | 1 | 18 | 4 | 14 |
| duck/Hunan/795/2002 | 2.1 | 18 | 3 | 15 |
| human/Indonesia/5/2005 | 2.1 | 20 | 4 | 16 |
| human/Indonesia/NIHRD11771/2011 | 2.1 | 18 | 4 | 14 |
| whooper-swan/Mongolia/244/2005 | 2.2 | 18 | 4 | 14 |
| bar-headed-goose/Qinghai/1A/2005 | 2.2 | 18 | 4 | 14 |
| turkey/Turkey/1/2005 | 2.2 | 19 | 4 | 15 |
| chicken/India/NIV33487/06 | 2.2 | 18 | 4 | 14 |
| human/Egypt/2321-NAMRU3/2007 | 2.2 | 20 | 4 | 16 |
| human/Egypt/3300-NAMRU3/2008 | 2.2 | 19 | 4 | 15 |
| human/Egypt/N03072/2010 | 2.2 | 21 | 4 | 17 |
| human/Anhui/1/2005 | 2.3 | 18 | 3 | 15 |
| Japanese-white-eye/HK/1038/2006 | 2.3 | 18 | 3 | 15 |
| duck/Laos/3295/2006 | 2.3 | 18 | 3 | 15 |
| common-magpie/HK/5052/2007 | 2.3 | 16 | 3 | 13 |
| chicken/HK/AP156/2009 | 2.3 | 18 | 3 | 15 |
| barn-swallow/HongKong/1161/2010 | 2.3 | 15 | 3 | 12 |
| human/Hubei/1/2010 | 2.3 | 16 | 3 | 13 |
| ck/Bangladesh/11rs1984-30/2011 | 2.3 | 18 | 3 | 15 |
| duck/Vietnam/NCVD1584/2012 | 2.3 | 16 | 3 | 13 |
| duck/Bangladesh/19097/2013 | 2.3 | 16 | 3 | 13 |
| human/Guizhou/1/2013 | 2.3 | 17 | 3 | 14 |
| human/Sichuan/26221/2014 (H5N6) | 2.3 | 14 | 3 | 11 |
| gyrfalcon/DC/41088-6/2014 (H5N8) | 2.3 | 13 | 3 | 10 |
| goose/Guiyang/337/2006 | 4 | 19 | 3 | 16 |
| chicken/Vietnam/NCVD-016/2008 | 7 | 20 | 2 | 18 |
| chicken/Vietnam/NCVD-03/2008 | 7 | 20 | 3 | 17 |
| environment/Hubei/950/2013 | 7 | 13 | 2 | 11 |

Note: Epitope binding affinity was measured by means as artificial neural network binding affinity (ANN IC50) to HLA-A. Epitopes with IC50 less than 50 nM were recognized as strong-binding, while an IC50 range between 50 to 500 nM was considered weak-binding. All viruses are HPAI H5N1 unless otherwise noted.

Table S5. Epitope conservancy analysis of 28 HPAI H5 clade-defining strains

| Clade-defining strains | Clades | No. of CD8+ T-cell epitopes | | |
| --- | --- | --- | --- | --- |
|  |  | **Total** | **Strong-Binding** | **Weak-Binding** |
| A/goose/Guangdong/1/1996 | 0 | 17 | 3 | 14 |
| A/duck/Vietnam/NCVD-16/2007 | 1.1 | 18 | 4 | 14 |
| A/chicken/Indonesia/BL/2003 | 2.1.1 | 21 | 4 | 17 |
| A/Indonesia/538H/2006 | 2.1.2 | 21 | 4 | 17 |
| A/chicken/Central Java/UT3091/2005 | 2.1.3 | 21 | 4 | 17 |
| A/chicken/East Java/UT6020/2006 | 2.1.3.1 | 21 | 4 | 17 |
| A/Indonesia/5/2005 | 2.1.3.2 | 20 | 4 | 16 |
| A/chicken/East Java/UT6045/2007 | 2.1.3.3 | 21 | 4 | 17 |
| A/bar-headed goose/Qinghai/3/2005 | 2.2 | 18 | 4 | 14 |
| A/chicken/Egypt/0836/2008 | 2.2.1.1 | 20 | 4 | 16 |
| A/Bangladesh/207095/2008 | 2.2.2 | 18 | 4 | 14 |
| A/duck/Hunan/127/2005 | 2.3.1 | 18 | 4 | 14 |
| A/chicken/Guangxi/2461/2004 | 2.3.2 | 20 | 4 | 16 |
| A/chicken/Guiyang/3055/2005 | 2.3.3 | 18 | 3 | 15 |
| A/Duck/Fujian/1734/2005 | 2.3.4 | 18 | 3 | 15 |
| A/environment/Guizhou/7/2009 | 2.3.4.1 | 16 | 3 | 13 |
| A/environment/Guizhou/2/2009 | 2.3.4.2 | 18 | 3 | 15 |
| A/chicken/Vietnam/NCVD-20/2007 | 2.3.4.3 | 20 | 3 | 17 |
| A/Ck/YN/115/2004 | 2.4 | 18 | 4 | 14 |
| A/chicken/Korea/es/2003 | 2.5 | 17 | 3 | 14 |
| A/chicken/Hong Kong/YU562/2001 | 3 | 16 | 3 | 13 |
| A/goose/Guangxi/914/2004 | 5 | 18 | 4 | 14 |
| A/blackbird/Hunan/1/2004 | 6 | 18 | 3 | 15 |
| A/chicken/Shanxi/2/2006 | 7 | 20 | 3 | 17 |
| A/chicken/Shanxi/10/2006 | 7.1 | 17 | 2 | 15 |
| A/chicken/Hebei/A-8/2009 | 7.2 | 21 | 3 | 18 |
| A/Ck/HK/YU777/2002 | 8 | 16 | 3 | 13 |
| A/duck/Guangxi/2775/2005 | 9 | 18 | 3 | 15 |

Note: Epitope binding affinity was measured by means as artificial neural network binding affinity (ANN IC50) to HLA-A. Epitopes with IC50 less than 50 nM were recognized as strong-binding, while an IC50 range between 50 to 500 nM was considered weak-binding. All viruses are HPAI H5N1 subtype.

Table S6. Study identified predicted epitopes screened against the experimentally determined epitopes database, Immunoepitope Database (IEDB)

| **# of distinct epitopes** | **Position on H5N1-HA protein** | **CD8+ T cell epitope** | **Experimentally Determined Epitopes** | | | | | | |
| --- | --- | --- | --- | --- | --- | --- | --- | --- | --- |
|  |  |  | **Source** | **B-cell** | | **CD8+ T cell** | | **CD4+ T cell** | |
|  |  |  |  | Epitope | IEDB ID | Epitope | IEDB ID | Epitope | IEDB ID |
| 1 | 18 | TIMEKNVTV | H5N1 |  |  |  |  | TEQVD**TIMEKNVTV**THAQ  EQVD**TIMEKNVTV**THAQ | 146023  128572 |
|  | 72 | NVPEWSYIV | H5N1 | **NVPEWSYIV**EKANPANDLCYPGNFNDYEELKHLLSR INHFEKIQ | 186786 |  |  | EFI**NVPEWSYIV**EKANPV | 145758 |
|  | 139 | GMPSFFRNV |  |  |  |  |  |  |  |
|  | 224  225 | GRMEFFWTI  RMDFFWTIL | H5N1 |  |  |  |  | RSKVNGQS**GRMEFFWTIL**  S**GRMEFFWTIL**KPNDAIN | 145994  146004 |
|  | 312 | VLATGLRNA |  |  |  |  |  |  |  |
|  | 332 | GLFGAIAGF | H1N1, H2N2  H5N1, H7N7,  H7N9 | RKKR**GLFGAIAGF**IE | 20836 | **GLFGAIAGF**I | 20837 | KKR**GLFGAIAGF**IEGGW | 97424 |
|  | 345 | GMVDGWYGF | H3N2 | GIFGAIAGFIENGWE**GMVDGWYGF**RHQNSEGTGQAADL | 141904 |  |  | AGFIENGWE**GMVDGWYGF**RHQNSEGTGQAADLKS | 1507 |
|  | 414 | KMEDGFLDV |  |  |  |  |  |  |  |
|  | 419 | FLDVWTYNA | H5N1  H3N2 |  |  |  |  | G**FLDVWTYNA**ELLVLMENER  ED**GFLDVWTYNA**ELLVLMEN | 19567  167894 |
|  | 431 | VLMENERTL | H5N1 |  |  | TYNAELL**VLMEN ERTL**DF | 173555 | ELL**VLMENERTL**DFHD | 13172 |
|  | 438 | TLDFHDSNV | H5N1 |  |  |  |  | LVLMENER**TLDFHDSNV**K | 131248 |
|  | 515 | YQILSIYST |  |  |  |  |  |  |  |
|  | 516 | QILSIYSTV | H5N1 |  |  | **QILSIYSTV** | 51101 |  |  |
|  | 517 | ILSIYSTVA |  |  |  |  |  |  |  |
|  | 523 | TVASSLALA |  |  |  |  |  |  |  |
|  | 532 | IMMAGLSLW |  |  |  |  |  |  |  |
|  | 537 | FLWMCSNGS | H1N1 |  |  |  |  |  |  |
| 2 | 43 | NLDGVKPLI | H5N1 | LC**NLDGVKPLI**LRDCSVAGWLLGNPMCDEFINVPE | 172022 |  |  | LC**NLDGVKPLI**LRDCSVAGWLLGNPMCDEFINVPE | 172022 |
|  | 103 | HLLSRINHL |  |  |  |  |  |  |  |
|  | 375 | AIDGVTNKV | H5N1 | KESTQK**AIDGVTNKV**NS | 162644 |  |  | TQK**AIDGVTNKV**NSIID | 130132 |
|  | 533 | MMAGLSLWM |  |  |  |  |  |  |  |
| 3 | 197 | YISVGTSTL | H5N1 |  |  |  |  | TT**YISVGTSTL**NQRLVPR | 146044 |
|  | 389 | KMNTQFEAV | H5N1 | QKAIDGVTNKVNSIID**KMNTQFEAV**GREFNNLERRI ENLNK | 97578 | **KMNTQFEAV** | 32378 | NSVIE**KMNTQFEAV**GKEFSN | 167981 |
|  | 531 | AIMMAGLSL |  |  |  |  |  |  |  |
| 4 | 527 | SLALAIMVA |  |  |  |  |  |  |  |
| 5 | 78 | YIVEKASPA |  |  |  |  |  |  |  |
|  | 529 | ALAIMVAGL |  |  |  |  |  |  |  |

Notes: **Empty cells**: Not found in the experimentally defined epitope database

**IEDB ID**: This identifier helps to retrieve the information from the [www.immuneepitope.org](http://www.immuneepitope.org) database.

**Summary of the table:**

In the current study we estimated a 46% (13/28) of predicted CD8+ T-cell epitopes in Table 2 Epitope 1 overlapped with the CD4+ T-cell epitopes and B-cell epitopes. Interestingly, twelve of 28 predicted CD8+ T-cell epitopes shown to be conserved and overlapped with the CD4+ T-cell epitopes. Overlapping T-cell responses to conserved epitope may significantly influence the course of influenza infection since the presence of CD4+ T-cell responses can greatly enhance and amplify the immune response of memory CD8+ T-cells and also regulate the production of neutralizing antibodies from B-lymphocytes (Johansen et al. 2004). These types of conserved and epitope-specific cross-immune response becomes crucial when a novel strain appears in the absence of pre-existing antibodies. We also found that one predicted CD8+ T-cell epitope (GLFGAIAGF) as commonly conserved across the sub-types of H1N1 (seasonal 1977-2009 and 2009 pandemic), H2N2, H5N1, H7N7, and H7N9; and the previously published experimental studies reported that GLFGAIAGF has potential to induce B- an T (CD8+ and CD4+) immune responses. This identified epitope may provide heterosubtypic protection.

**References:**

Johansen P, Stamou P, Tascon RE, Lowrie DB, Stockinger B. CD4+ T cells guarantee optimal competitive fitness of CD8+ memory T cells. Eur J Immunol 2004;34(January (1)):91–7.

Figure S1. Heat map predicting the capability of vaccine candidates to induce preexisting immunity in humans for currently circulating HPAI H5 strains


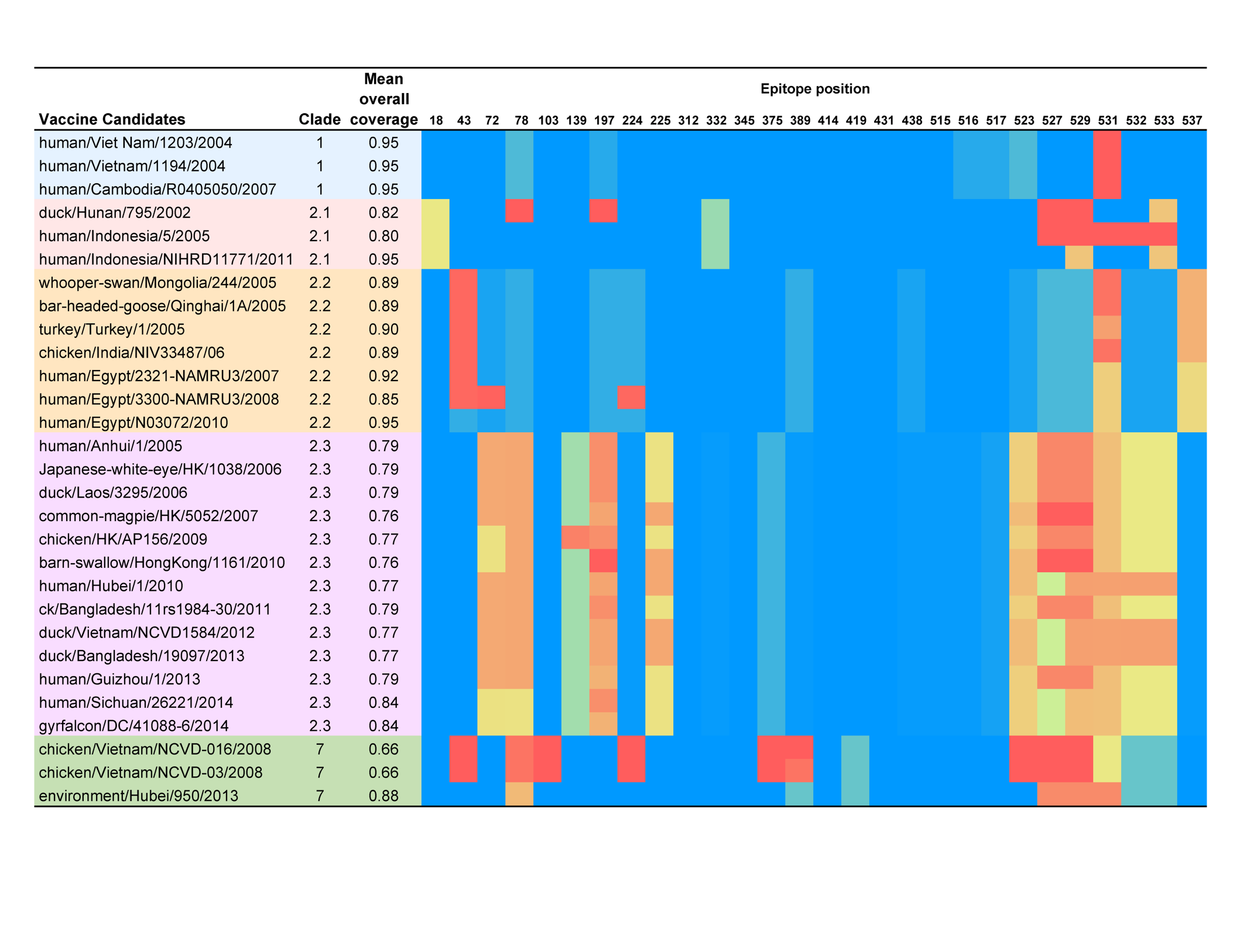


0.00 0.75 1.00


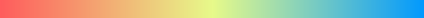
 Legend:

Clade-specific epitope profile was reported as the proportion of H5 strains in each clade that were resembled by each epitope in this vaccine candidate. Mean epitope coverage, as the measure for overall potential of one vaccine candidate to induce preexisting immunity in humans, was calculated from averaging the proportions in different epitope positions.
